# Urban Nature and Public Health: How Nature Exposure and Sociocultural Background Relate to Depression Risk

**DOI:** 10.3390/ijerph18189689

**Published:** 2021-09-14

**Authors:** Kristen Jakstis, Leonie K. Fischer

**Affiliations:** Institute of Landscape Planning and Ecology, University of Stuttgart, 70174 Stuttgart, Germany; leonie.fischer@ilpoe.uni-stuttgart.de

**Keywords:** mental health, public health, depression risk, urban nature exposure, urban gardening behavior, sociodemographic characteristics, immigration history

## Abstract

As the world’s population becomes more urbanized, there is an associated decrease in nature exposure and a rise in noncommunicable diseases, including depression. Previous cross-sectional studies examining urban nature exposure and depression have reported favorable associations. However, many of these studies rely primarily on nature exposure metrics that measure the intensity of nature exposure, while other dimensions of urban nature exposure remain understudied. Therefore, in a cross-sectional, questionnaire-based case study targeting a general urban population (*n* = 282), we examined the relationship between two less commonly studied urban nature exposure variables (i.e., gardening behavior and greenspace visit frequency) and depression risk while also considering sociocultural background (multivariate logistic regression model). Results indicated that being a gardener was significantly associated with a reduced odds of being at risk of depression and that having a family migration history, but not a self-migration history, was associated with increased odds of being at risk of depression. In the examination of neighborhood socialization frequency and depression risk, we did not determine any significant association. The results of this study, therefore, highlight the importance of considering both people’s sociocultural backgrounds and urban nature exposure in more detail to help plan for and support healthier cities in the future.

## 1. Introduction

Exposure to the natural environment has long been associated with a variety of positive physiological and psychological health outcomes. These include, among others, improved mental health [1,2], stress reduction and recovery [3,4,5], reduced prevalence of obesity [6,7] and even lower all-cause mortality [8,9]. However, two-thirds of the world population is projected to live in urban areas by 2050 [10] and nature exposure, and possibly the health benefits derived from it, is reduced with increasing urbanization [11,12]. 

Meanwhile, the prevalence of noncommunicable diseases, including mental health disorders, is increasing with urbanization [13,14]. Worldwide, mental health challenges contribute to economic losses of up to one trillion U.S. dollars annually, and specifically in the WHO European Region, mental health challenges are the top cause of disability and are in the top three causes of overall disease burden [15]. The most prevalent of these mental health challenges in Europe is depression, with an estimated 44.3 million sufferers [15]. Furthermore, depression has been associated with comorbidities, including diabetes, cardiovascular disease, chronic respiratory diseases and cancer [16]. 

Studies examining the relationship between urban nature exposure and depression risk or depressive symptoms have generally reported positive findings [11,17,18,19]. Three domains of pathways—mitigation, restoration and instoration—have been proposed to explain how nature exposure may support this relationship and other mental health outcomes [20,21,22]. Mitigation describes the reduction of harmful environmental stressors, such as air pollution; restoration describes the improvement of restoring capacities, for example, through stress reduction; instoration describes the improvement of building capacities, such as improved social health and increased physical activity [20]. Indeed, there is evidence that the relationship between nature exposure and mental health may be explained via a combination of these three pathways. 

However, the instoration pathway has received less attention than mitigation and restoration in urban nature exposure–mental health studies [20]. This remains true considering both social factors and depression, despite a well-established link between these factors outside of nature exposure studies [23]. For example, a diverse range of social factors, such as social support, social networks and social connectedness, have been associated with depression risk [24,25,26]. In many cases, such social health benefits may be at least partially obtained within people’s neighborhoods [27], and neighborhood and community social factors have been previously associated with depression risk [28,29]. Despite these findings, results remain mixed regarding neighborhood social factors in nature exposure–depression studies [17,30,31]. For instance, Cox and colleagues found evidence of both increased social cohesion and reduced depression with increasing nearby nature exposure [17], while others found social factors played a very minor role in the nature exposure–depression relationship [30]. These mixed results and the disproportionately low focus on social health outcomes and factors in urban nature exposure studies, necessitates further research into the relationships among urban nature exposure, social factors and depression.

A variety of methods are used in public health studies to assess the two overarching dimensions of urban nature exposure: amount and characteristics. The amount of nature exposure can be quantified by considering its duration, frequency and intensity. For instance, Shanahan and colleagues (2016) used a nature-dose framework that included each of these three aspects to examine the amount of nature exposure in relation to depression among other health outcomes [18]. Despite examples such as this, most observational studies examining depression risk commonly use metrics that primarily characterize the intensity of urban nature exposure such as the Normalized Difference Vegetation Index (NDVI) [30,32], tree canopy cover [33] or the ratio of greenspace within a buffer [19]. Metrics such as these can easily quantify greenness over large sample areas using remote sensing methods and are, therefore, often employed in urban nature–depression risk studies. However, these metrics derived from remote sensing methods can only measure the intensity of urban nature exposure. The other two aspects concerning nature exposure amount (i.e., duration and frequency) have been less extensively studied and have mixed results regarding depression risk [11,18].

The second dimension of urban nature exposure that can be considered in mental health studies are nature exposure characteristics. Urban nature exposure characteristics include, for example, types of nature-based activities and the intentionality (i.e., incidental vs. purposeful) of nature exposure [20]. One such purposeful nature-based activity is gardening. Favorable associations have been found not only between gardening activities and depression itself [34], but also factors related to depression risk such as stress [35] and social cohesion or interaction [36]. However, most studies that examine the relationship between gardening and depression either specifically target groups of gardeners [34,36] or specifically vulnerable groups of society, such as those already diagnosed with depression or other mental health disorders [37,38,39], people with disabilities [40,41], refugees [42] or the elderly [43,44]. Often, studies involve interventions, such as horticultural therapy, that have defined treatment goals and are specifically designed to treat people with depression or other health issues [35,37,38,39,44,45]. This is not the same as ‘typical’ gardening, which involves gardening in or around one’s own home or in community or allotment gardens, usually without the structure of guided activities or intentional therapy. To our knowledge, few cross-sectional studies have examined the relationship between gardening behavior and depression risk in a general urban population (but see e.g., [43] with a focus on allotment gardeners and [40] that focuses on people with disabilities but does not preferentially target gardeners).

In addition to various measures of nature exposure, it is also important to consider the possible effects of sociocultural backgrounds on depression risk. This is because sociodemographic and socioeconomic factors often relate more strongly to health than nature exposure, for instance green space availability [46]. For example, age has long been a well-accepted risk factor for depression due to the cumulative effects of other health issues attributed to aging [47]. However, the relationship between other sociocultural factors, such as migration history and depression risk, is less clear. There are many potential stressors, such as language barriers, reduced social networks and loss of previous profession or social status, associated with migration that could lead to mental health issues such as depression [48]. While not unanimous, some studies have found migrants and those with a family migration history are at greater risk of depression than their non-migrant counterparts [49,50,51]. Additionally, studies examining the relationship between specifically generational migration history and depression risk have yielded mixed results [50,51]. For example, some studies found first-generation migrants have a greater depression risk compared to second-generation migrants and non-migrants [50], while others found second-generation migrants are more at risk of depression than first-generation migrants [51]. Furthermore, despite possible benefits of urban nature exposure for people with a migration history [42,52,53] and garden-based intervention projects targeted towards migrant populations [53], there are a lack of studies examining generational migration history and its association with nature exposure in the general urban population.

In this cross-sectional study targeting a general urban population, we, therefore, examined the relationship between urban nature exposure and depression risk, while considering sociocultural factors, such as neighborhood socialization frequency and migration history. The multicultural city of Stuttgart, Germany, where approximately 45% of residents have a migration history [54], was used as a model city and 364 respondents of diverse sociocultural backgrounds surveyed, of which 282 cases serve as the basis of this study. Urban nature exposure was assessed using understudied aspects of exposure characteristics and amount, namely gardening behavior and greenspace visit frequency. We hypothesized that (1) urban nature exposure and (2) neighborhood social interaction are associated with lower odds of being at risk of depression, and that (3) depression risk, urban nature exposure and neighborhood social interaction differ among participants according to migration history.

## 2. Materials and Methods

### 2.1. Field Survey

Our field survey was conducted in Stuttgart, Germany from 25 July to 2 October 2020. Stuttgart is Germany’s sixth largest city with over 609,000 residents and a population density of 3067 residents per square kilometer [54]. Approximately 45% of these residents have a migration history [54]. To target the general urban population of Stuttgart, we distributed our questionnaire into postboxes of residents and by approaching pedestrians in two neighborhoods. Printed media rather than online techniques were used to include many different people, for example those with lower German or English comprehension skills or those without easy internet access. Additionally, selected neighborhoods in Stuttgart had slightly higher proportions of self-migrants (both 33%) than the average for Stuttgart (25%) [54]. With this higher migrant population, we aimed to capture a more representative sample of Stuttgart residents, as residents with a migration history are often under-represented in general population surveys [55]. Questionnaires were distributed to pedestrians on both weekdays and weekends covering the 12 h period from 8 am to 8 pm. When pedestrians were approached, the applicable COVID-19 hygiene regulations were followed by trained staff (i.e., minimum two-meter distance, masks, regular sanitation of hands and equipment). In total 364 questionnaires were returned, the majority (84%) of which were from pedestrians with a 56% acceptance rate. 

### 2.2. Questionaire 

To explore our hypotheses, we assembled a three-part questionnaire available in English and German that addressed participants’ (1) habits and preferences pertaining to urban nature, greenspaces and neighborhood socialization, (2) current health status and (3) information on their sociocultural background. All questions pertaining to urban greenspace, nature exposure and sociocultural background were derived from existing and previously validated questionnaires [56,57], with few changes in wording to improve clarity. Responses and comments from a small test sample (*n* = 10), comprising male and female participants, aged 20 to 58 years, with six different nationalities, were used to verify the clarity of the fully assembled questionnaire used in this study. 

In part one of the questionnaire, an urban nature exposure characteristic, gardening behavior, was assessed with the yes–no question: “Do you garden (on balcony, windowsill, etc.)?”. Additionally, an aspect of urban nature exposure amount, greenspace visit frequency, was addressed with the closed multiple-choice question: “In the last two weeks, how often did you visit greenspaces in your city?”. Neighborhood socialization frequency was also assessed using a closed multiple-choice question: “In the last two weeks, how often did you socialize with your neighbors?”. 

In part two of the questionnaire, the standardized World Health Organization-Five Well-Being Index (WHO-5) was used to collect information on depression risk. The WHO-5 consists of five positively phrased statements that respondents rate from 0 (at no time) to 5 (all the time) concerning the applicability of each statement to themselves considering the past two weeks [58]. 

Finally, in part three of the questionnaire, information on respondents’ sociocultural background was collected using simply phrased questions regarding age, gender, educational background, employment status and migration history. To address age, participants were asked to simply write-in their response. Gender was assessed using a closed multiple-choice question with three responses: “male,” “female” and “other”. Educational background, employment status and migration history were assessed using closed multiple-choice questions. For example, migration history was assessed using the question: “Were you, your parents or your grandparents born in a country other than Germany?”. It should be noted that although the term *migration* specifies temporary movement and *immigration* the intent of permanent residency, we henceforth use the terms *migration/migrant/migration history* to describe both migration and immigration, as we did not specify intent in the questionnaire. Original survey questions and responses are provided in Table A1. 

### 2.3. Data Preparation 

The original survey questions and responses from each part of the questionnaire were transcribed into variables to be used for analyses, the questionnaires digitalized, and the resulting digitalized data spot checked for accuracy. Repeats from participants’ that answered the questionnaire more than once, those from respondents under 18, or those that were missing responses for any of the variables of interest were removed from analyses resulting in 282 observations used for the analyses at hand.

Urban nature exposure variables (i.e., gardening behavior and greenspace visit frequency) and neighborhood socialization frequency were derived as categorical predictor variables from part one of the questionnaire (see Table 1 for variables and their respective levels). Risk of depression was assessed and adapted into a bivariate response variable using the WHO-5 score from part two of the questionnaire. Following standard procedure, responses to the WHO-5 were summed and multiplied by four to calculate a score out of 100. A WHO-5 cut-off score of ≤50 has a sufficiently high sensitivity to be used as a valid screening tool for depression [59], and was, therefore, considered as “at risk of depression” in the study at hand. Variables describing sociocultural background (i.e., migration history, educational background, gender and age), all categorical excepting age, were obtained from part three of the questionnaire. Finally, levels of categorical variables that had very small response rates were combined or dropped where necessary to allow for reliable statistical analyses. The derivation of the variables used in analyses are provided in Table A1. 

### 2.4. Statistical Analyses 

Descriptive statistics were first conducted to characterize the study population (see “3.1. Sample Description”). Next, to consider both urban nature exposure variables together in addition to sociocultural factors that may influence this relationship, a multivariate logistic regression model with depression risk as the response was created. As some predictor variables had more than two levels, generalized variance inflation factors (GVIF) adjusted for degree of freedom were calculated, and a threshold of adjusted GVIF <2 used to assess and avoid multicollinearity. The best fit model was selected using the calculated Akaike Information Criterion (AIC) as guidance and included five predictor variables: gardening behavior, greenspace visit frequency, neighborhood socialization frequency, migration history and age. Odds ratios and their 95% confidence intervals were then calculated and plotted using this best fit logistic regression model. To examine the bivariate relationships between migration history and urban nature exposure and social interaction individually, Pearson’s Chi-squared tests were conducted and bivariate mosaic plots visually examined to assess the directionality of these relationships. Cramer’s V was then calculated to estimate effect sizes for the significant associations indicated by the Chi-squared tests. Finally, a stratified analysis according to migration history examining the significance of associations between gardening behavior and depression risk was also conducted for additional background information; the estimated *p*-values resulting from these Fisher’s Exact Tests are presented in Table A2. All statistical analyses were conducted in R version 4.0.3 [60]. 

## 3. Results

### 3.1. Sample Description 

The average WHO-5 score describing the depression risk of participants was 64.2, similar to the German average of 64.7 according to the 2016 European Quality of Life Survey [61]. In the sample population, 19.5% of participants were considered at risk of depression according to the WHO-5 ≤50 cutoff score. Concerning the two urban nature exposure variables, 68% of respondents were gardeners and 60% reported visiting greenspaces in their city several times per week. Regarding neighborhood socialization frequency, 66% of respondents reported socializing with neighbors at least once per week. Thirty-seven percent of respondents reported having a migration history stemming from at least 38 countries and five continents, with 43% of those identifying as self-migrants (self-migration history) and 57% as the children or grandchildren of migrants (family migration history). There was a large age range of adult participants spanning 18 to 93 years. The majority of participants were university-educated, with 62% of respondents holding a higher-education degree (Table 1). 

### 3.2. Urban Nature Exposure and Depression Risk 

**Figure 1:**
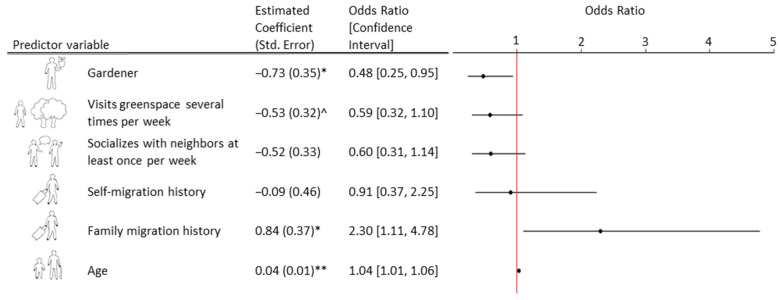
Estimated coefficients and their respective standard errors calculated from the multivariate logistic regression model with ‘at risk of depression’ as the response (*n* = 282). The odds ratios and their confidence intervals calculated from these estimated coefficients are also provided and visually represented with an odds plot on the right. Two nature exposure variables, ‘gardener’ (baseline: ‘non-gardener’) and ‘visits greenspace several times per week’ (baseline: ‘visits greenspace once per week or less’), are reported. The other predictor variables included in the model are ‘socializes with neighbors at least once per week’ (baseline: ‘socializes with neighbors less than per week’), ‘self-’ and ‘family migration history’ (baseline: ‘no migration history’) and ‘age.’ Marginally significant results (*p* < 0.1) are denoted with (^), significant results (*p* < 0.05) with (*) and highly significant results (*p* < 0.01) with (**).

The best fit logistic regression model used for this analysis included two urban nature exposure predictor variables—gardening behavior and greenspace visit frequency. Results of this model indicated that being a gardener was significantly (*p* = 0.036) associated with a reduced odds of being at risk of depression, with an odds ratio of 0.48 (Figure 1). While visiting greenspaces several times per week followed this same directional trend, this finding was only marginally significant (*p* = 0.098; Figure 1). 

### 3.3. Neighborhood Social Interaction and Depression Risk 

The best fit logistic regression model used for this analysis included one social predictor variable—neighborhood socialization frequency. Results of this model indicated that socializing with neighbors at least once per week was not significantly associated with depression risk (Figure 1).

### 3.4. Migration History

#### 3.4.1. Depression Risk 

The best fit logistic regression model used for this analysis included migration history as a predictor variable. According to this model, the relationship between migration history and depression risk varied among those with a self-migration history and those with a family migration history. While there was not a significant relationship between self-migration history and depression risk, having a family migration history was significantly associated (*p* = 0.025, Figure 1) with increased odds of being at risk of depression.

#### 3.4.2. Urban Nature Exposure and Neighborhood Social Interaction 

**Figure 2:**
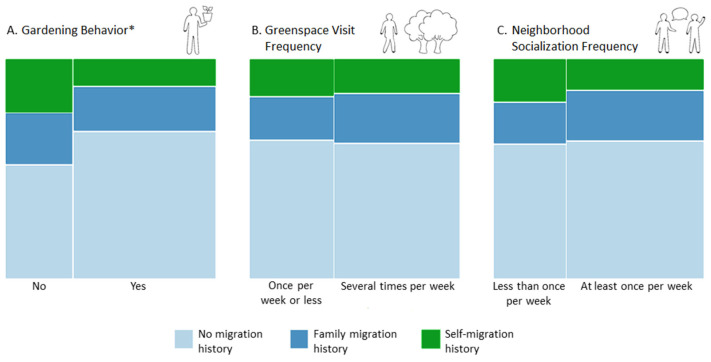
Mosaic plots depicting bivariate relationships between migration history and gardening behavior, greenspace visit frequency and neighborhood socialization frequency. The width of each bar represents the proportion of respondents (*n* = 282) in the respective group. The height of the individual-colored sections within each bar represents the proportion of respondents in each group according to the levels within the variable migration history (i.e., no migration history, family migration history and self-migration history). The bivariate relationships depicted in the plots are between migration history and (**A**) gardening behavior, (**B**) greenspace visit frequency and (**C**) neighborhood socialization frequency. Significant bivariate associations according to Chi-squared analyses are denoted with (*) in the individual heading of each plot.

Chi-squared analyses and mosaic plots were used to explore bivariate relationships between migration history and both urban nature exposure and neighborhood socialization frequency. Chi-squared analyses indicated a significant association between migration history and gardening behavior (*p* = 0.014, Figure 2), with a Cramer’s V of 0.17, indicating a small to medium effect size. Subsequent visual examination of this bivariate mosaic plot suggests those with a migration history are less often gardeners than their German counterparts without a migration history. There were no significant associations found between migration history and greenspace visit frequency nor migration history and neighborhood socialization frequency. 

## 4. Discussion

In this cross-sectional study targeting a general urban population, we found that being a gardener was significantly associated with a decreased odds of being at risk of depression, while neither greenspace visits nor neighborhood socialization frequency was significantly associated with depression risk. Previous studies have indicated a favorable relationship between urban nature exposure and depression [17,18,19,30,32,33]; however, many of these studies focus primarily on the intensity of exposure [19,30,32,33]. We, therefore, considered understudied aspects of urban nature exposure characteristics (i.e., gardening behavior) and amount (i.e., greenspace visit frequency) to better understand more specific aspects of nature exposure in relation to depression risk. Additionally, sociocultural factors were also considered in analyses to explore how these relationships may be affected by the unique background of residents.

### 4.1. Urban Nature Exposure and Depression Risk

Our results support our first hypothesis that urban nature exposure is associated with decreased odds of being at risk of depression. However, of the two urban nature exposure variables selected for this study, only gardening behavior (being a gardener) was significantly associated with decreased odds of being at risk of depression. Generally, there is a large evidence base that supports the positive effect of horticultural therapy on depression [37,38,39,41,44]. Different to our cross-sectional study, many of these are intervention studies that explore the effectiveness of horticultural therapy on at-risk populations, such as the elderly or those with pre-existing mental health disorders [37,39,44]. For example, a study conducted on clinically depressed participants found horticultural therapy significantly lowered depression scores and this improvement was maintained three months after the intervention [39]. It must be noted, however, that horticultural therapy is not synonymous with gardening, as it is an intentionally designed intervention that includes features, such as patient evaluation and goal setting, with the unique aim to improve health [45]. 

Although horticultural therapy and gardening are distinct nature-based activities, many studies examining participants’ motivations for gardening suggest that people often garden for therapeutic purposes [36,62]. For example, McFarland and colleagues (2018) found 92.1% of respondents provided motivations for gardening that can be considered therapeutic [62]. Similarly, a study focused on allotment gardening in urban Japan conducted by Soga and colleagues (2017), observed ‘taking a mental break’ was the most common motivation for gardening [36]. Additionally, multiple therapeutic effects and factors related to depression have been associated with gardening, such as improvements in self-esteem and mood [34] and stress reduction [63].

In relation to gardening behavior, other potential underlying motivations or habits of respondents should also be considered. Interestingly, educational background may also affect motivations to garden, as a recent study found that participants with less formal education were more likely to garden for recreational and health reasons and spent twice as long gardening per week as those with more formal education [64]. This is important to consider within the context of our study because, while we did not include educational background in our final multivariate model, the majority of our sample population attended university, which may have affected our results. Nevertheless, the results of our study support the idea that ‘general’ gardening, outside of any professional therapeutic context, may have a therapeutic or protective effect, as being a gardener was associated with decreased odds of being at risk of depression. However, the cross-sectional design of this study does not allow for the establishment of causation. Therefore, it is also possible that those at risk of depression are less likely to garden. This is feasible, as neglecting interests and hobbies is often a symptom of depression [65]. 

To our knowledge, there are few other cross-sectional studies that have examined the relationship between gardening behavior of a general urban population and depression risk per se. One notable exception conducted by Wood and colleagues (2016) found gardeners experienced significantly less depression than non-gardeners [34]. However, it should be noted that this study specifically targeted allotment gardeners and not other types of gardeners, nor the general public and was part of a larger case-control study. Nonetheless, our results are in line with those from Wood and colleagues (2016) in addition to other cross-sectional studies that examined gardening behavior and health outcomes that encompass depression, such as mental health [36] and well-being [66,67]. While mental health and well-being are not the same as depression risk per se, questionnaires used to assess these health outcomes often include depression or symptoms of depression as an aspect within their assessment [68,69]. It should be noted that we relied upon responses to the WHO-5 to assess depression risk and did not ask if participants were medically diagnosed with depression. However, as many people with depression are likely undiagnosed [70,71] and the WHO-5 has sufficiently high sensitivity to be used as a valid screening tool for depression [59], we believe this was an effective method to determine depression risk.

While being a gardener was significantly associated with a decreased odds of being at risk of depression in our study, frequently visiting greenspaces was only marginally significant. Contrary to our results, previous studies have indicated that the amount of urban nature exposure quantified using various urban greenspace metrics is significantly associated with depression risk and symptoms [19,30,31,32,72]. However, many of these studies used greenspace metrics that quantify the intensity of nature exposure amount rather than its other dimensions, such as duration or frequency [19,30,32,33]. Studies that examined specifically frequency of urban nature exposure and depression have yielded mixed results [11,18]. For example, Cox and colleagues (2018) found that frequent greenspace visits in urbanized populations was associated with a reduction in depression [11], while another study indicated that duration of greenspace visits but not frequency was associated with lower rates of depression [18]. As our results indicated only a marginally significant association between greenspace visit frequency and depression risk, it is possible that duration or intensity of exposure may have provided more insight concerning the amount of urban nature exposure and depression risk for our sample. 

### 4.2. Neighborhood Social Interaction and Depression Risk

Our results do not support our second hypothesis that frequency of neighborhood social interaction is associated with decreased odds of being at risk of depression. Although the beneficial relationship between positive social factors and depression is well-established outside of urban nature studies and are often considered to be potential mediators in the relationship between nature exposure and depression, studies have yielded mixed results [17,23,31]. One potential reason that socialization frequency was not associated with depression risk in our study could be attributed to the selection of our social variable — frequency of socialization with neighbors. We specifically focused on a neighborhood social factor because neighborhood and community social factors, such as social cohesion, have been previously associated with depression [28,73]. However, inconsistent results regarding neighborhood social factors and depression risk have also been reported [74] and it is possible that our focus was too narrow to adequately represent social interaction in a way that would significantly affect depression risk in our sample. Additionally, while we focused on the frequency of social interaction with neighbors, results from some studies suggest that quality of social interactions, rather than quantity, is more protective of depression risk [26,75]. In this context, it should also be noted that the study at hand was conducted in the summer and early autumn of 2020 within the first year of the COVID-19 pandemic. During this time, social interactions with neighbors, for example while gardening in yards or on balconies, may have been a primary source of social contact when other social situations were still limited. However, insights into possible behavior shifts during the COVID-19 pandemic are just beginning to be explored, and participants may have had reduced social contacts during this time or even associated negative emotions with social interaction due to fear of infection [76], despite low infection rates during this phase of the pandemic in the study area [77]. 

### 4.3. Depression Risk, Urban Nature Exposure and Neighborhood Social Interaction according to Migration History

Our results offer mixed support for our third hypothesis that depression risk, urban nature exposure and neighborhood social interaction differ among participants according to migration history. More specifically, we found that having a family migration history, but not self-migration history, was significantly associated with increased odds of being at risk of depression. Our results are contrary to some studies that have found first-generation migrants have a greater depression risk than second-generation or non-migrants [50]. Indeed, there are potential stressors such as language barriers, reduced social networks and loss of previous profession or social status associated with migration that could lead to mental health issues, such as depression [48]. However, there are also factors that may have negative health consequences for specifically second-generation migrants, for example, experiencing emotional conflicts between their familial culture and the culture of their birth country (see e.g., [51]). In support of this, and similar to the results of our own study, Ruiz-Castell and colleagues (2017) found that the prevalence of depressive symptoms was highest in second-generation migrants, followed by first-generation migrants and then non-migrants [51]. Additionally, first-generation migrants may sometimes experience a health boost, but then this health benefit tends to decrease the longer the migrant is in the new country and second-generation migrants typically have a worse health status than first-generation migrants [78]. 

Moreover, in partial support of our third hypothesis, bivariate analyses between migration history and urban nature exposure and neighborhood socialization frequency yielded mixed results. No significant associations in relation to migration history and greenspace visit frequency nor frequency of socialization with neighbors were indicated. However, our results did reveal a significant association between migration history and gardening behavior with a small to medium estimated effect size. Visual assessment of this plotted relationship suggested that a smaller proportion of participants with a migration history were gardeners compared with non-gardeners. Although we did not examine motivations behind gardening behavior, there are several factors that could contribute to the negative association between migration history and gardening behavior. For example, it is possible that people with a migration history are less likely to have time or space at home to garden and may not live near or be informed about community gardens. A study targeting Mexican women who migrated to the USA found they performed fewer gardening activities than women from their home state in Mexico. They cited being less confident and knowledgeable about gardening and a lack of adequate space as reasons for not gardening in their new country of residence [79]. While the context of this study differs from the one at hand, it is possible that these factors may also contribute the relationship between migration history and gardening behavior seen here. 

### 4.4. Strengths and Limitations

There are several notable strengths of this study. First, we considered understudied aspects of urban nature exposure amount and characteristics in relation to depression risk. Many previous cross-sectional studies focused primarily on the intensity of urban nature exposure [19,30,32,33], rather than other aspects of nature exposure characteristics or amount. By using ‘gardening behavior’ and ‘greenspace visit frequency’ as exposure metrics, the results of this study provide a unique insight into the relationship between urban nature exposure and depression risk. Additionally, this is one of few studies that has examined the relationship between gardening behavior and depression risk in a general urban population. While mental health benefits of targeted gardening programs, such as horticultural therapy, are extensively studied [37,38,39,41,44], there are few studies examining gardening behavior and depression risk more generally. In the few cross-sectional studies that have examined, this relationship differs from ours, as they targeted populations with disabilities [40], specific groups of gardeners [34], or did not examine depression risk per se but rather mental health or well-being more generally [36,66]. Finally, studies examining aspects of gardening behavior and migration history are diverse and include research on potential benefits of gardening for people with a migration history [42,80], attitudes towards gardening [79], effects of gardening intervention projects [42,53] and even species composition of gardens in relation to migration history [64]. To our knowledge, however, we are among the first studies that examined if migration history is associated with gardening behavior in a general urban population. Based on our initial insights, the relationship between migration history and urban nature exposure, including gardening behavior, could be addressed in even more detail in future studies.

There are also several limitations to this study. First, this study’s cross-sectional design only provides a snapshot of the actual situation and does not allow for causal inference. Another potential limitation is that we only included a limited number of predictor variables in the multivariate model due to the sample size. Additionally, some predictor variables, such as neighborhood socialization frequency and greenspace visit frequency, were restructured to have two levels, which likely led to the loss of some statistical variance but allowed more predictor variables to be included in the multivariate model. While this helped ensure the reliability of the model by avoiding overfitting, including predictor variables with more levels as well as additional predictor variables would have allowed for a more fully adjusted model, potentially offering more insight. Including more predictors describing socioeconomic status would have been particularly relevant, as socioeconomic status may be associated with the amount of time and space (at home) available to urban residents for gardening. It should also be noted that although educational background, one indicator of socioeconomic status, was not included in the multivariate model, the majority of our sample was university educated, which could be a potential source of bias in this study. Additionally, while we did consider greenspace visit frequency as an understudied urban nature exposure metric, we did not consider the motivation behind greenspace visits (i.e., purposeful versus incidental visits). Future studies could include the characteristic of intentionality of greenspace visits in combination with frequency for a more robust consideration of this aspect of urban nature exposure. Furthermore, while we included a social factor in our analyses, we did not conduct a formal mediation analysis among urban nature exposure, neighborhood social interaction and depression risk. Therefore, although our results offer insight into individual aspects of this relationship, this pathway was not examined in its entirety. Finally, we also recognize the rather large errors for migration history in the logistic regression model and resulting odds ratios. This suggests high within-group variation that could be attributed to the fact that we only differentiated between generational migration history (i.e., self-migration history versus family migration history) despite migration being a broad term that includes people of many distinct backgrounds with diverse experiences. Future studies should consider more specific aspects of migration history to better understand its role in the relationship between urban nature exposure and depression risk. 

## 5. Conclusions

In an urbanizing world, nature exposure is decreasing whilst the prevalence of mental health disorders, such as depression, is increasing. Depression is the most prevalent mental health disorder in Europe, and as many people with depression are left untreated or even undiagnosed [70,71,81], a better understanding of urban nature exposure–depression risk relationships could contribute towards large mental health benefits. Three main pathways—mitigation, restoration and instoration—have been proposed to describe how urban nature exposure may affect mental health [20,21,22]. While there is a growing evidence base supporting these pathways, disproportionately few studies have examined aspects related to the instoration pathway concerning urban nature exposure, social factors and depression risk. Additionally, although urban nature exposure is complex in itself, most studies focus primarily on the intensity of nature exposure in relation to depression risk. Therefore, in a cross-sectional study targeting a general urban population we examined the relationship between understudied aspects of urban nature exposure and depression risk while also considering sociocultural factors. Our results indicated that being a gardener was associated with decreased odds of being at risk of depression and that gardening behavior and depression risk varied according to generational migration history. While our results are not causal, they support the idea that to help ensure healthier cities, public initiatives that enable gardening for all citizens, including people across diverse backgrounds and those without private garden access, should be supported. The results of this study further highlight the importance of considering both people’s sociocultural backgrounds and urban nature exposure in more detail to help plan for and support healthier cities in the future. 

## Figures and Tables

**Table 1:** Descriptive statistics for the sample population (*n* = 282) of variables considered in analyses. All values reported as ‘percentage of the sample population,’ with absolute counts of sample population provided next to the percentage in parentheses, unless otherwise noted. For the derivation of these variables, see Table A1.

Variable		Level	Percent Sample Population
1. Depression risk		At risk of depression (WHO-5 ≤ 50)	19.5 (*n* = 55)
		Not at risk of depression (WHO-5 > 50)	80.5 (*n* = 227)
2. Gardening behavior	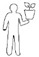	Gardener	68.1(*n* = 192)
		Non-gardener	31.9 (*n* = 90)
3. Greenspace visit frequency	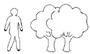	Visits greenspace several times a week	59.9 (*n* = 169)
		Visits greenspace once a week or less	40.1(*n* = 113)
4. Neighborhood socialization frequency	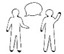	Socializes with neighbors at least once a week	65.6 (*n* = 185)
		Socializes with neighbors less than once a week	34.4 (*n* = 97)
5. Migration history	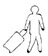	No migration history (German)	62.8 (*n* = 177)
		Self-migration history	16.0 (*n* = 45)
		Family migration history	21.3 (*n* = 60)
6. Educational background	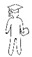	Secondary school completed	19.1 (*n* = 54)
		Vocational education completed	18.4 (*n* = 52)
		University education completed	62.4 (*n* = 176)
7. Gender	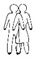	Female	55.4 (*n* = 157)
		Male	44.3 (*n* = 125)
8. Age	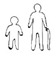	Age (years)	Range: 18–93 Mean: 36.2 (SD: 13.4)

## Data Availability

The data presented in this study are available on request from the corresponding author. The data are not publicly available to privacy considerations.

## Appendix A

**Table A1:** Summary table of variables and their derivation from the original survey questions. Repeat surveys and those that were missing responses or contained “I do not know” responses to any of the survey questions listed here were removed before analyses. The resulting level of the merged categories ‘none,’ ‘primary’ and ‘other’ for the variable ‘educational background’ was also dropped due to its low sample size (*n* = 7). Those seven observations are, therefore, not considered in the summaries of the other predictor variables, resulting in a total of 282 observations used in analyses.

Variable	Original Survey Question	Original Survey Responses	Level Name for Analyses	N for Original Levels	Merged Levels for Analyses	New Level Name of Merged Categories for Analyses	N for Each New Level
Depression risk	WHO-5: Over the last two weeks: 1. I have felt cheerful and in good spirits 2. I have felt calm and relaxed 3. I have felt active and vigorous 4. I woke up feeling fresh and rested 5. My daily life has been filled with things that interest me	Responses per WHO-5 question: All the time (5) Most of the time (4) More than half of the time (3) Less than half of the time (2) Some of the time (1) At no time (0)	Originally continuous so no level name (raw score out of 25, multiplied by 4 to yield score out of 100.)	282	Cut-off score of WHO-5 ≤ 50 considered ‘At risk of depression.’	At risk of depression Not at risk of depression	55 227
Gardening behavior	Do you garden (on balcony, windowsill, etc.)?	Yes No	Yes No	192 90	Yes No	Gardener Non-gardener	192 90
Greenspace visit frequency	In the last two weeks, how often did you visit green spaces in your city?	Several times a week Once a week Less than once a week Never I do not know	Several Once Less Never DK	169 85 19 9 NA	Several Once + Less + Never	Visits greenspace several times per week Visits greenspace once per week or less	169 113
Neighborhood socialization frequency	In the last two weeks, how often did you socialize with your neighbors?	Several times a week Once a week Less than once a week Never I do not know	Several Once Less Never DK	99 86 59 38 NA	Several + Once Less + Never	Socializes with neighbors at least once per week Socializes with neighbors less than once per week	185 97
Migration history	Were you, your parents or your grandparents born in a country other than Germany?	No, we were all born in Germany. Yes, I was born in another country, specifically in: Yes, at least one of my parents was born in another country, specifically in: Yes, at least one of my grandparents was born in another country, specifically in: I do not know.	German Self Parent_foreign Grandparent_foreign DK	177 45 43 17 NA	German Self Parent_foreign + Grandparent_foreign	German Self-migration history Family migration history	177 45 60
Gender	Gender:	Male Female Other	Male Female Other	125 157 0	Male Female	Male Female	125 157
Age	Age:	Write-in	Continuous; no levels	282	Age	Age	282
Educational background	What is the highest level of schooling you have completed?	No formal schooling Primary school completed Secondary school completed Vocational education completed University education completed Other, specifically:	None Primary Secondary Vocational University Other	1 2 54 52 176 4	None + Primary + other Secondary Vocational University	Dropped Secondary Vocational University	Dropped 54 52 176

**Table A2:** Estimated p-values according to Fischer’s Exact Tests for the bivariate association between depression risk and gardening behavior stratified by migration history.

Migration History Subset	Fisher’s Exact *p*-value
No migration history (*n* = 117)	0.07
Self-migration history (*n* = 45)	0.46
Family migration history (*n* = 60)	0.56

## References

[1] Banay R.F., James P., Hart J.E., Kubzansky L.D., Spiegelman D., Okereke O.I., Spengler J.D., Laden F. (2019). Greenness and Depression Incidence among Older Women. Environ. Health Perspect..

[2] Helbich M., Klein N., Roberts H., Hagedoorn P., Groenewegen P.P. (2018). More Green Space Is Related to Less Antidepressant Prescription Rates in the Netherlands: A Bayesian Geoadditive Quantile Regression Approach. Environ. Res..

[3] Hedblom M., Gunnarsson B., Iravani B., Knez I., Schaefer M., Thorsson P., Lundström J.N. (2019). Reduction of Physiological Stress by Urban Green Space in a Multisensory Virtual Experiment. Sci. Rep..

[4] Herrera R., Markevych I., Berger U., Genuneit J., Gerlich J., Nowak D., Schlotz W., Vogelberg C., von Mutius E., Weinmayr G. (2018). Greenness and Job-Related Chronic Stress in Young Adults: A Prospective Cohort Study in Germany. BMJ Open.

[5] Thompson C.W., Roe J., Aspinall P., Mitchell R., Clow A., Miller D. (2012). More Green Space Is Linked to Less Stress in Deprived Communities: Evidence from Salivary Cortisol Patterns. Landsc. Urban Plan..

[6] Villeneuve P.J., Jerrett M., Su J.G., Weichenthal S., Sandler D.P. (2018). Association of Residential Greenness with Obesity and Physical Activity in a US Cohort of Women. Environ. Res..

[7] Huang W.-Z., Yang B.-Y., Yu H.-Y., Bloom M.S., Markevych I., Heinrich J., Knibbs L.D., Leskinen A., Dharmage S.C., Jalaludin B. (2020). Association between Community Greenness and Obesity in Urban-Dwelling Chinese Adults. Sci. Total Environ..

[8] James P., Hart J.E., Banay R.F., Laden F. (2016). Exposure to Greenness and Mortality in a Nationwide Prospective Cohort Study of Women. Environ. Health Perspect..

[9] Crouse D.L., Pinault L., Balram A., Hystad P., Peters P.A., Chen H., van Donkelaar A., Martin R.V., Ménard R., Robichaud A. (2017). Urban Greenness and Mortality in Canada’s Largest Cities: A National Cohort Study. Lancet Planet. Health.

[10] United Nations, Department of Economic and Social Affairs, Population Division (2019). World Urbanization Prospects 2018: Highlights.

[11] Cox D.T.C., Shanahan D.F., Hudson H.L., Fuller R.A., Gaston K.J. (2018). The Impact of Urbanisation on Nature Dose and the Implications for Human Health. Landsc. Urban Plan..

[12] Soga M., Gaston K.J. (2016). Extinction of Experience: The Loss of Human–Nature Interactions. Front. Ecol. Environ..

[13] Okkels N., Kristiansen C.B., Munk-Jørgensen P., Sartorius N. (2018). Urban Mental Health: Challenges and Perspectives. Curr. Opin. Psychiatry.

[14] Hoare E., Jacka F., Berk M. (2019). The Impact of Urbanization on Mood Disorders: An Update of Recent Evidence. Curr. Opin. Psychiatry.

[15] (2019). Fact Sheet—Mental Health. https://web.archive.org/web/20210721100601/https://www.euro.who.int/__data/assets/pdf_file/0004/404851/MNH_FactSheet_ENG.pdf.

[16] Cohen A. (2017). Addressing Comorbidity between Mental Disorders and Major Noncommunicable Diseases: Background Technical Report to Support. Implementation of the WHO European Action Plan. for the Prevention and Control. of Noncommunicable Diseases 2016–2025.

[17] Cox D.T.C., Shanahan D.F., Hudson H.L., Fuller R.A., Anderson K., Hancock S., Gaston K.J. (2017). Doses of Nearby Nature Simultaneously Associated with Multiple Health Benefits. Int. J. Env. Res. Public Health.

[18] Shanahan D.F., Bush R., Gaston K.J., Lin B.B., Dean J., Barber E., Fuller R.A. (2016). Health Benefits from Nature Experiences Depend on Dose. Sci. Rep..

[19] Lee H.J., Lee D.K. (2019). Do Sociodemographic Factors and Urban Green Space Affect Mental Health Outcomes among the Urban Elderly Population?. Int. J. Environ. Res. Public Health.

[20] Beute F., Andreucci M.B., Davies Z., Glanville J., Keune H., Marselle M., O’Brien L.A., Olszewska-Guizzo A., Remmen R., Russo A. (2020). Type and Characteristics of Urban. and Peri-Urban. Green Spaces Having an Impact on Human Mental Health and Wellbeing: A Systematic Review.

[21] Bratman G.N., Anderson C.B., Berman M.G., Cochran B., de Vries S., Flanders J., Folke C., Frumkin H., Gross J.J., Hartig T. (2019). Nature and Mental Health: An Ecosystem Service Perspective. Sci. Adv..

[22] Markevych I., Schoierer J., Hartig T., Chudnovsky A., Hystad P., Dzhambov A.M., de Vries S., Triguero-Mas M., Brauer M., Nieuwenhuijsen M.J. (2017). Exploring Pathways Linking Greenspace to Health: Theoretical and Methodological Guidance. Environ. Res..

[23] Holt-Lunstad J., Smith T.B., Layton J.B. (2010). Social Relationships and Mortality Risk: A Meta-Analytic Review. PLoS Med..

[24] Santini Z.I., Koyanagi A., Tyrovolas S., Mason C., Haro J.M. (2015). The Association between Social Relationships and Depression: A Systematic Review. J. Affect. Disord..

[25] Santini Z.I., Jose P.E., Cornwell E.Y., Koyanagi A., Nielsen L., Hinrichsen C., Meilstrup C., Madsen K.R., Koushede V. (2020). Social Disconnectedness, Perceived Isolation, and Symptoms of Depression and Anxiety among Older Americans (NSHAP): A Longitudinal Mediation Analysis. Lancet Public Health.

[26] Werner-Seidler A., Afzali M.H., Chapman C., Sunderland M., Slade T. (2017). The Relationship between Social Support Networks and Depression in the 2007 National Survey of Mental Health and Well-Being. Soc. Psychiatry Psychiatr. Epidemiol..

[27] Lane A.P., Hou Y., Hooi Wong C., Yuen B. (2020). Cross-Sectional Associations of Neighborhood Third Places with Social Health among Community-Dwelling Older Adults. Soc. Sci. Med..

[28] Miao J., Wu X., Sun X. (2019). Neighborhood, Social Cohesion, and the Elderly’s Depression in Shanghai. Soc. Sci. Med..

[29] Dawson C.T., Wu W., Fennie K.P., Ibañez G., Cano M.Á., Pettit J.W., Trepka M.J. (2019). Perceived Neighborhood Social Cohesion Moderates the Relationship between Neighborhood Structural Disadvantage and Adolescent Depressive Symptoms. Health Place.

[30] Gascon M., Sánchez-Benavides G., Dadvand P., Martínez D., Gramunt N., Gotsens X., Cirach M., Vert C., Molinuevo J.L., Crous-Bou M. (2018). Long-Term Exposure to Residential Green and Blue Spaces and Anxiety and Depression in Adults: A Cross-Sectional Study. Environ. Res..

[31] Liu Y., Wang R., Xiao Y., Huang B., Chen H., Li Z. (2019). Exploring the Linkage between Greenness Exposure and Depression among Chinese People: Mediating Roles of Physical Activity, Stress and Social Cohesion and Moderating Role of Urbanicity. Health Place.

[32] Hystad P., Payette Y., Noisel N., Boileau C. (2019). Green Space Associations with Mental Health and Cognitive Function. Environ. Epidemiol..

[33] Browning M.H.E.M., Lee K., Wolf K.L. (2019). Tree Cover Shows an Inverse Relationship with Depressive Symptoms in Elderly Residents Living in U.S. Nursing Homes. Urban For. Urban Green..

[34] Wood C.J., Pretty J., Griffin M. (2016). A Case–Control Study of the Health and Well-Being Benefits of Allotment Gardening. J. Public Health.

[35] Vujcic M., Tomicevic-Dubljevic J., Grbic M., Lecic-Tosevski D., Vukovic O., Toskovic O. (2017). Nature Based Solution for Improving Mental Health and Well-Being in Urban Areas. Environ. Res..

[36] Soga M., Cox D., Yamaura Y., Gaston K., Kurisu K., Hanaki K. (2017). Health Benefits of Urban Allotment Gardening: Improved Physical and Psychological Well-Being and Social Integration. Int. J. Environ. Res. Public Health.

[37] Kim K.-H., Park S.-A. (2018). Horticultural Therapy Program for Middle-Aged Women’s Depression, Anxiety, and Self-Identify. Complement. Ther. Med..

[38] Ghanbari S., Jafari F., Bagheri N., Neamtolahi S., Shayanpour R. (2015). Study of the Effect of Using Purposeful Activity (Gardening) on Depression of Female Resident in Golestan Dormitory of Ahvaz Jundishapur University of Medical Sciences. J. Rehabil. Sci..

[39] Gonzalez M.T., Hartig T., Patil G.G., Martinsen E.W., Kirkevold M. (2010). Therapeutic Horticulture in Clinical Depression: A Prospective Study of Active Components: Therapeutic Horticulture in Clinical Depression. J. Adv. Nurs..

[40] Wilson J.F., Christensen K.M. (2011). The Relationship between Gardening and Depression among Individuals with Disabilities. J. Ther. Hortic..

[41] Triguero-Mas M., Anguelovski I., Cirac-Claveras J., Connolly J., Vazquez A., Urgell-Plaza F., Cardona-Giralt N., Sanyé-Mengual E., Alonso J., Cole H. (2020). Quality of Life Benefits of Urban Rooftop Gardening for People With Intellectual Disabilities or Mental Health Disorders. Prev. Chronic. Dis..

[42] Gerber M.M., Callahan J.L., Moyer D.N., Connally M.L., Holtz P.M., Janis B.M. (2017). Nepali Bhutanese Refugees Reap Support Through Community Gardening. Int. Perspect. Psychol..

[43] Masuya J., Ota K., Mashida Y. (2014). The Effect of a Horticultural Activities Program on the Psychologic, Physical, Cognitive Function and Quality of Life of Elderly People Living in Nursing Homes. Int. J. Nurs. Clin. Pract..

[44] Park S.-A., Lee A.-Y., Son K.-C., Lee W.-L., Kim D.-S. (2016). Gardening Intervention for Physical and Psychological Health Benefits in Elderly Women at Community Centers. HortTechnology.

[45] Relf D., Dorn S. (1995). Horticulture: Meeting the Needs of Special Populations. HortTechnology.

[46] Kabisch N. (2019). The influence of socio-economic and socio-demographic factors in the association between urban green space and health. Biodiversity and Health in the Face of Climate Change.

[47] Roberts R.E., Kaplan G.A., Shema S.J., Strawbridge W.J. (1997). Prevalence and Correlates of Depression in an Aging Cohort: The Alameda County Study. J. Gerontol. B Psychol. Sci. Soc. Sci..

[48] Morawa E., Erim Y. (2014). Acculturation and Depressive Symptoms among Turkish Immigrants in Germany. Int. J. Environ. Res. Public Health.

[49] Davison K.M., Lung Y., Lin S., Tong H., Kobayashi K.M., Fuller-Thomson E. (2019). Depression in Middle and Older Adulthood: The Role of Immigration, Nutrition, and Other Determinants of Health in the Canadian Longitudinal Study on Aging. BMC Psychiatry.

[50] Sieberer M., Maksimović S., Ersöz B., Machleidt W., Ziegenbein M., Calliess I.T. (2012). Depressive Symptoms in First-and Second-Generation Migrants: A Cross-Sectional Study of a Multi-Ethnic Working Population. Int. J. Soc. Psychiatry.

[51] Ruiz-Castell M., Kandala N.-B., Perquin M., Bocquet V., Kuemmerle A., Vögele C., Stranges S. (2017). Depression Burden in Luxembourg: Individual Risk Factors, Geographic Variations and the Role of Migration, 2013–2015 European Health Examination Survey. J. Affect. Disord..

[52] Gentin S., Pitkänen K., Chondromatidou A.M., Præstholm S., Dolling A., Palsdottir A.M. (2019). Nature-Based Integration of Immigrants in Europe: A Review. Urban Urban Green.

[53] Hartwig K.A., Mason M. (2016). Community Gardens for Refugee and Immigrant Communities as a Means of Health Promotion. J. Community Health.

[54] Stuttgart in Zahlen. https://web.archive.org/web/20210804132928/https://www.stuttgart.de/service/statistik-und-wahlen/stuttgart-in-zahlen.php.

[55] Méndez M., Ferreras M., Cuesta M. (2013). Immigration and general population surveys in Spain: The CIS surveys. Surveying Ethnic Minorities and Immigrant Populations: Methodological Challenges and Research Strategies.

[56] Fischer L.K., Honold J., Cvejić R., Delshammar T., Hilbert S., Lafortezza R., Nastran M., Nielsen A.B., Pintar M., van der Jagt A.P.N. (2018). Beyond Green: Broad Support for Biodiversity in Multicultural European Cities. Glob. Environ. Chang..

[57] Gopal D., Fischer L. (2021). Streetscapes as Surrogate Greenspaces During COVID-19?. Front. Sustain. Cities.

[58] WHO. https://web.archive.org/web/20210628142711/https://www.psykiatri-regionh.dk/who-5/Documents/WHO-5%20questionaire%20-%20English.pdf.

[59] Topp C.W., Østergaard S.D., Søndergaard S., Bech P. (2015). The WHO-5 Well-Being Index: A Systematic Review of the Literature. Psychother. Psychosom..

[60] R Core Team R: A Language and Environment for Statsitcal Computing. https://www.R-project.org/.

[61] Europäische Erhebung zur Lebensqualität—Datenvisualisierung. https://web.archive.org/web/20210804133125/https://www.eurofound.europa.eu/de/data/european-quality-of-life-survey.

[62] McFarland A., Waliczek T.M., Etheredge C., Sommerfeld Lillard A.J. (2018). Understanding Motivations for Gardening Using a Qualitative General Inductive Approach. HortTechnology.

[63] Hawkins J.L., Thirlaway K.J., Backx K., Clayton D.A. (2011). Allotment Gardening and Other Leisure Activities for Stress Reduction and Healthy Aging. HortTechnology.

[64] Philpott S.M., Egerer M.H., Bichier P., Cohen H., Cohen R., Liere H., Jha S., Lin B.B. (2020). Gardener Demographics, Experience, and Motivations Drive Differences in Plant Species Richness and Composition in Urban Gardens. Ecol. Soc..

[65] Symptoms—Clinical Depression. https://web.archive.org/web/20210904033453/https://www.nhs.uk/mental-health/conditions/clinical-depression/symptoms/.

[66] Koay W.I., Dillon D. (2020). Community Gardening: Stress, Well-Being, and Resilience Potentials. Int. J. Environ. Res. Public. Health.

[67] Chalmin-Pui L.S., Griffiths A., Roe J., Heaton T., Cameron R. (2021). Why Garden?—Attitudes and the Perceived Health Benefits of Home Gardening. Cities.

[68] The Warwick-Edinburgh Mental Wellbeing Scale (WEMWBS). https://web.archive.org/web/20210804133325/https://warwick.ac.uk/fac/sci/med/research/platform/wemwbs/.

[69] Lundin A., Hallgren M., Theobald H., Hellgren C., Torgén M. (2016). Validity of the 12-Item Version of the General Health Questionnaire in Detecting Depression in the General Population. Public Health.

[70] Ko J.Y., Farr S.L., Dietz P.M., Robbins C.L. (2015). Depression and Treatment Among U.S. Pregnant and Nonpregnant Women of Reproductive Age, 2005–2009. J. Womens Health.

[71] Farr S.L., Bitsko R.H., Hayes D.K., Dietz P.M. (2010). Mental Health and Access to Services among US Women of Reproductive Age. Am. J. Obstet. Gynecol..

[72] Song H., Lane K.J., Kim H., Kim H., Byun G., Le M., Choi Y., Park C.R., Lee J.-T. (2019). Association between Urban Greenness and Depressive Symptoms: Evaluation of Greenness Using Various Indicators. Int. J. Environ. Res. Public. Health.

[73] Mair C., Roux A.V.D., Golden S.H., Rapp S., Shea S. (2015). Change in Neighborhood Environments and Depressive Symptoms in New York City: The Multi-Ethnic Study of Atherosclerosis. Health Place.

[74] Wang R., Xue D., Chen H., Qiu Y. (2018). The Relationship between Urbanization and Depression in China: The Mediating Role of Neighborhood Social Capital. Int. J. Equity Health.

[75] Nezlek J.B., Imbrie M., Shean G.D. (1994). Depression and Everyday Social Interaction. J. Per. Soc. Psychol..

[76] Williams S.N., Armitage C.J., Tampe T., Dienes K. (2020). Public Perceptions and Experiences of Social Distancing and Social Isolation during the COVID-19 Pandemic: A UK—Based Focus Group Study. BMJ Open.

[77] Infektionen und Todesfälle in Baden-Württemberg (Archiv). https://web.archive.org/web/20210804133745/https://www.baden-wuerttemberg.de/de/service/aktuelle-infos-zu-corona/archiv-infektionen-und-todesfaelle-in-baden-wuerttemberg/infektionen-und-todesfaelle-in-baden-wuerttemberg-3-quartal-2020/.

[78] Alegría M., Álvarez K., DiMarzio K. (2017). Immigration and Mental Health. Curr. Epidemiol. Rep..

[79] Bellows A.C., Alcaraz G.V., Vivar T. (2009). Gardening as a Tool to Foster Health and Cultural Identity in the Context of International Migration: Attitudes and Constraints in a Female Population. Acta Hortic..

[80] Agustina I., Beilin R. (2012). Community Gardens: Space for Interactions and Adaptations. Procedia Soc. Behav. Sci..

[81] World Health Organization (WHO) Regional Office for Europe Depression in Europe: Facts and Figures. https://web.archive.org/web/20210804133946/https://www.euro.who.int/en/health-topics/noncommunicable-diseases/mental-health/news/news/2012/10/depression-in-europe/depression-in-europe-facts-and-figures.

